# Inflamm-ageing or inflammasom-ageing as independent events

**DOI:** 10.18632/aging.104108

**Published:** 2020-09-28

**Authors:** Mario D. Cordero, Jesús Ruiz-Cabello

**Affiliations:** 1 Cátedra de Reproducción y Genética Humana del Instituto para el Estudio de la Biología de la Reproducción Humana (INEBIR), Universidad Europea del Atlántico (UNEATLANTICO), Fundación Universitaria Iberoamericana (FUNIBER), Sevilla, Spain; 2 CIC biomaGUNE, San Sebastian-Donostia, Spain; 3 IKERBASQUE, Basque Foundation for Science, Bilbao, Spain; 4 CIBER de Enfermedades Respiratorias (CIBERES), Madrid, Spain; 5 Universidad Complutense Madrid, Madrid, Spain

**Keywords:** inflamm-ageing, inflammasome, NLRP3, inflammation, ageing

**Figure 1:**
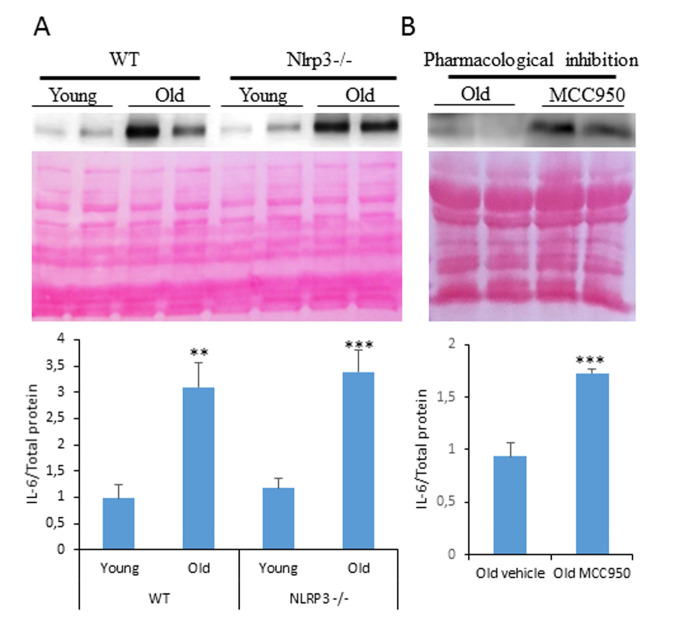
**Changes in the autophagy and inflammation observed in ovaries from young and old mice.** (**A**) Western blot analysis with representative blot of IL-6 levels in cardiac tissue of 3 and 20-mo-old WT and Nlrp3 -/-. (**B**) Western blot analysis showing the protein expression of inflammatory markers IL-6 in cardiac tissues of old mice after MCC950 treatment. n= 4 mice per group and age. Densitometric analysis is shown as means ± SD. ***P < 0.001, **P < 0.01, young *vs* old mice.

Inflammasomes are multiprotein complexes that are formed and activated after exposure to danger signals with the activation of caspase-1 causing the maturation and release of IL-1β and IL-18. In the formation of inflammasome complexes, several cytosolic pattern recognition receptors (PRRs) act as sensors of pathogen-associated molecular patterns (PAMPS) and damage-associated molecular patterns (DAMPS) [1]. NLRP3-inflammasome is the most described complex consisting of a leucine-rich repeat (LRR) domain, a central nucleotide-binding oligomerization domain (NOD), and an amino-terminal pyrin domain (PYD), which primarily interacts with apoptosis-associated speck-like protein containing a CARD (ASC) [1]. The main interest of the NLRP3-inflammasome is the activity in response to a wide variety of stimuli, such as endogenous danger signals such as extracellular adenosine triphosphate (ATP), uric acid crystals (MSU), and amyloid-β fibrils, free cholesterol, ROS [2]. Many of these stimuli are elevated during ageing, and are implicated in age-related diseases, so the activation of the NLRP3 inflammasome appears to be involved in the production of age-related inflammation. Accordingly, NLRP3-inflammasome has been associated with inflamm-ageing, a low-grade sterile chronic inflammation that has been described as a progressive event during biological ageing with accumulation of pro-inflammatory mediators [3]. Inflammasomes are suspected to play a role in inflammaging, however, their exact contribution to this process remains unknown [4]. Our recent study showed that the genetic deletion of Nlrp3 in mice prolongs lifespan and improves healthspan by attenuating the multiple age-related degenerative changes associated with cardiac and metabolic ageing [5]. Deletion of NLRP3-inflammasome protected aged mice from age-related increase in insulin resistance, reduced IGF-1 and leptin/adiponectin ratio levels. Furthermore, NLRP3 ablation reduced cardiac damage with protection from age-dependent PR interval prolongation, which is associated with atrial fibrillation from cardiovascular ageing and reduced telomere shortening. Interestingly, these KO mice showed inhibition of the PI3K/AKT/mTOR pathway, with consequent improved autophagy flux compared to older wild type mice, and conserved Nampt-mediated NAD^+^ levels with increased SIRT1 protein expression. So, based on this, the role of the NLRP3-inflammasome during ageing and the inflamm-ageing is very evident. However, the complexity of the inflammasomes shows an independent role of these with respect to the canonical inflammatory pathways. Nlrp3 -/- mice have shown similar levels of other cytokines compared to their littermates controls. Youm et al., showed increased levels of IL-6 in serum from aged Nlrp3 -/- and WT mice [6]. In our recent work, we showed levels similar to serum levels of IL-6, IL-8, TNF, and increased levels of IL-6 protein in cardiac tissues from old mice, but there was a greater increase in heart tissues from old KO mice than WT mice and a similar increase in serum from WT and KO mice (Figure 1A). Interestingly, pharmacological inhibition of Nlrp3 in old mice also shows increased cardiac IL-6 expression (Figure 1B). IL-6, IL-8 and TNF, and their receptors, are upregulated in aged tissues and cells [4]. These findings raise the possibility that the loss of NLRP3 does not affect the age-related increase in other inflammatory pathways and inflamm-ageing, but with the protective effect of the age-related changes that confers the inflammasome an important and independent role in cardiac and metabolic ageing. Therefore, we propose the possibility of an independent aspect of the NLRP3-inflammasome from the inflamm-ageing that could be associated with a new inflammasom-ageing in which the only inhibition of the NLRP3-inflammasome can prevent various aspects related to age.

Further investigation focused on the specific role of NLRP3-inflammasome in cardiac ageing will be required to address these issues and would open a new pharmacological target for the use of the specific NLRP3 inhibitor with respect to anti-inflammatory treatments. We invite to the scientific community to discuss this possible new topic.
